# A New Histone Deacetylase Inhibitor Enhances Radiation Sensitivity through the Induction of Misfolded Protein Aggregation and Autophagy in Triple-Negative Breast Cancer

**DOI:** 10.3390/cancers11111703

**Published:** 2019-11-01

**Authors:** Hui-Wen Chiu, Ya-Ling Yeh, Sheng-Yow Ho, Yuan-Hua Wu, Bour-Jr Wang, Wei-Jan Huang, Yuan-Soon Ho, Ying-Jan Wang, Li-Ching Chen, Shih-Hsin Tu

**Affiliations:** 1 Graduate Institute of Clinical Medicine, College of Medicine, Taipei Medical University, Taipei 11031, Taiwan; leu3@tmu.edu.tw; 2 Division of Nephrology, Department of Internal Medicine, Shuang Ho Hospital, Taipei Medical University, Taipei 11031, Taiwan; 3 Department of Environmental and Occupational Health, College of Medicine, National Cheng Kung University, Tainan 70101, Taiwan; linn7627@hotmail.com (Y.-L.Y.); wuyh@mail.ncku.edu.tw (Y.-H.W.); yjwang@mail.ncku.edu.tw (Y.-J.W.); 4 Department of Radiation Oncology, Chi Mei Medical Center, Tainan 71004, Taiwan; shengho@seed.net.tw; 5 Graduate Institute of Medical Sciences, Chang Jung Christian University, Tainan 71101, Taiwan; 6 Department of Radiation Oncology, National Cheng Kung University Hospital, College of Medicine, National Cheng Kung University, Tainan 70101, Taiwan; 7 Department of Occupational and Environmental Medicine, National Cheng Kung University Hospital, Tainan 70101, Taiwan; pochih.wang@msa.hinet.net; 8 Department of Cosmetic Science and Institute of Cosmetic Science, Chia Nan University of Pharmacy and Science, Tainan 71710, Taiwan; 9 Graduate Institute of Pharmacognosy, Taipei Medical University, Taipei 11031, Taiwan; wjhuang@tmu.edu.tw; 10 TMU Research Center of Cancer Translational Medicine, Taipei Medical University, Taipei 11031, Taiwan; hoyuansn@tmu.edu.tw (Y.-S.H.); lcchen@tmu.edu.tw (L.-C.C.); 11 Graduate Institute of Medical Sciences, College of Medicine, Taipei Medical University, Taipei 11031, Taiwan; 12 Department of Laboratory Medicine, Taipei Medical University Hospital, Taipei 11031, Taiwan; 13 School of Medical Laboratory Science and Biotechnology, College of Medical Science and Technology, Taipei Medical University, Taipei 11031, Taiwan; 14 Division of Breast Surgery, Department of Surgery, Taipei Medical University Hospital, Taipei 11031, Taiwan; 15 Taipei Cancer Center, Taipei Medical University, Taipei 11031, Taiwan; 16 Department of Surgery, School of Medicine, College of Medicine, Taipei Medical University, Taipei 11031, Taiwan

**Keywords:** histone deacetylase inhibitor, aggresome, radiation, autophagy, triple-negative breast cancer

## Abstract

Radiation therapy (RT) is one of the main treatments for triple-negative breast cancer (TNBC). However, many patients experience RT failure due to the metastatic potential of RT and the radiation resistance of several cancers. Histone deacetylase inhibitors (HDACis) can serve as radiosensitizers. In this study, we investigated whether a novel HDACi, TMU-35435, could reinforce radiosensitivity through the induction of misfolded protein aggregation and autophagy in TNBC. Significantly enhanced toxicity was found for the combination treatment compared with TMU-35435 or irradiation (IR) treatment alone in TNBC cells. The combination treatment induced misfolded protein aggregation and TMU-35435 inhibited the interaction of HDAC6 with dynein. Furthermore, the combined treatment induced endoplasmic reticulum (ER) stress but did not trigger apoptosis. In addition, the combination treatment caused autophagic cell death. Tumor growth in the mouse of model orthotopic breast cancer was suppressed by the combination treatment through the induction of ER stress and autophagy. These findings support the future evaluation of the novel HDACi TMU-35435, as a potent radiosensitizer in TNBC.

## 1. Introduction

Breast cancer is the most diagnosed cancer and a major cause of death in women worldwide [1]. Triple-negative breast cancer (TNBC) is described by the lack of estrogen receptors, progesterone receptors, or human epidermal growth factor receptor 2 [2]. Many reports have shown that TNBC is associated with poor prognosis and is more likely than other breast cancer to recur locally and metastasize to the lung and brain during 3–5 years after diagnosis [2,3]. Radiation therapy (RT) is one of the primary treatments for TNBC. RT reduces the risk of local recurrence and increases overall survival in in situ and infiltrating breast cancer [4]. However, due to the metastatic potential of RT and individual variation in radiosensitivity, many patients experience RT failure, which leads to cancer relapse and metastasis [5]. Therefore, it is necessary to develop novel strategies that can strengthen the effectiveness of radiotherapy.

The autophagy–lysosomal pathway and the ubiquitin–proteasome system (UPS) are two primary self-digestive mechanisms for cellular proteins. The UPS is the selective degradation pathway for the proteolysis of misfolded or short-lived proteins. In this pathway, misfolded proteins binding with ubiquitin are degraded by proteasome [6]. Previous studies have demonstrated that excessive misfolded or unfolded proteins in the endoplasmic reticulum (ER) cause ER stress and induce unfolded protein response (UPR) pathways [7]. ER stress and UPR pathways in cancer therapies provide very potential for the development of novel anti-cancer strategies [8]. If the stress is too severe, ER stress can also trigger cell death [9,10]. Evidence indicates that ER stress is a trigger of apoptosis and autophagy [8,11,12]. Furthermore, Williams et al. indicated that proteasome inhibitor induced ER stress and cell death in human colon cancer cells [9]. Therefore, the UPS may regulate ER stress. Recent evidence shows that the aggresome is an alternative system to the proteasome for the degradation of polyubiquitinated unfolded/misfolded proteins [13]. Aggresome formation finally causes autophagic clearance, which degrades many substrates (or cargoes) via the lysosomal pathway [14]. Histone deacetylase 6 (HDAC6) plays an important role in aggresomal protein degradation. HDAC6 binds both dynein and polyubiquitinated proteins for transport to aggresomes [15]. Previously the authors reported that HDAC6 inhibitor panobinostat caused ER stress and autophagy in TNBC cells [16]. These findings suggest that the UPS is closely related to autophagy.

Autophagy is a protein degradation mechanism that recycles damaged organelles and long-lived proteins by transporting them in autophagosomes to lysosomes for degradation [17]. When exposed to adverse environments, such as nutrient starvation or hypoxia, cells induce autophagy to maintain their longevity [18]. In recent years, targeting autophagy to strengthen current treatments in several cancers, including breast cancer, has shown promising results [18,19,20]. Although autophagy plays a critical role in cancer therapy, the role of autophagy in controlling cancer cell death or survival is still being debated. Previous research has shown that ER stress in the breast cancer cells with defective apoptosis mechanism increases irradiation (IR)-induced autophagy and reinforces radiosensitivity. The activation of autophagy through UPR mechanisms may serve as a potential radiosensitization strategy to strengthen the killing efficiency of RT in breast cancer cells [21]. Our previous studies found that a new histone deacetylase inhibitor (HDACi), TMU-35435, enhanced etoposide cytotoxicity by the proteasomal degradation of DNA-dependent protein kinase catalytic subunit (DNA-PKcs) in TNBC [22]. TMU-35435 had antitumor and enhanced activity of the DNA demethylation reagent against human non-small cell lung cancer [23]. Therefore, we hypothesized that TMU-35435 can sensitize TNBC cells to IR. We tested this hypothesis by exploring the impacts of TMU-35435 combined with IR on the UPS, ER stress and autophagy in TNBC cell lines. We examined whether TMU-35435 enhanced sensitivity to IR in vitro and in vivo. Our observations provide novel perceptions into the mechanisms underlying TMU-35435-mediated radiosensitization that may be important for developing strategies to improve the efficiency of TNBC to RT.

## 2. Results

### 2.1. Cytotoxic Effects of TMU-35435 and IR Treatment Separately or in Combination on TNBC Cells

**Figure 1:**
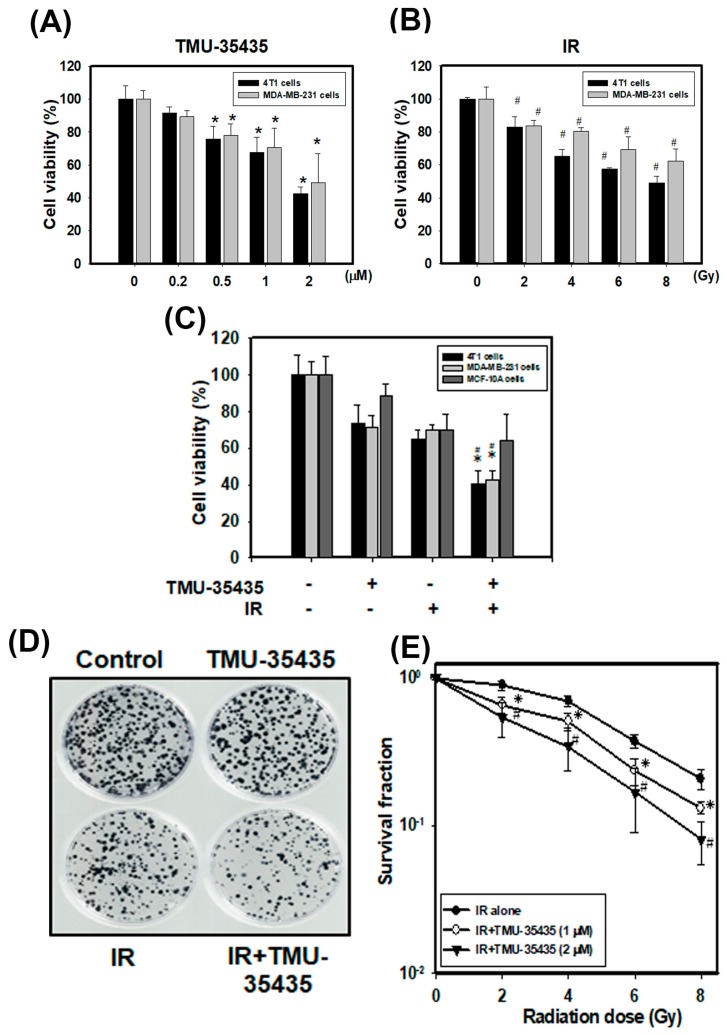
Cytotoxic effects of TMU-35435 and IR in MDA-MB-231 and 4T1 cells. (**A**) The cell viability at different concentrations. The cells were incubated with 0.2, 0.5, 1, or 2 μM TMU-35435 for 24 h. * *p* < 0.05, TMU-35435 versus control. (**B**) The cell viability at different doses. The cells were treated with 2, 4, 6, or 8 Gy of IR for 24 h. # *p* < 0.05, IR versus control. (**C**) Cell viability effects of TMU-35435 (1 μM) and IR (4 Gy) for 24 h. * *p* < 0.05, TMU-35435 versus combination treatment. # *p* < 0.05, IR versus combination treatment. (**D**) Clonogenic assays in 4T1 cells treated with IR (4 Gy) and/or TMU-35435 (1 μM). Colonies (containing ≥50 cells) were stained with crystal violet solution. (**E**) IR dose–response survival curves of 4T1 cells with or without TMU-35435. * *p* < 0.05, IR alone versus IR + TMU-35435 (1 μM). # *p* < 0.05, IR alone versus IR alone versus IR + TMU-35435 (2 μM).

Our previous report indicated that TMU-35435 concentration-dependently increased acetylation of histone H4 and tubulin (a nonhistone protein) in MDA-MB-231 and 4T1 cells [22]. TMU-35435 showed better inhibitory effects on total HDAC activity and isoform-specific HDAC activity, including HDAC6 activity, than did suberoylanilide hydroxamic acid (SAHA) [23], which was the first Food and Drug Administration (FDA)-approved HDACi for the treatment of lymphoma [24]. The viability was analyzed at different doses or concentrations of IR or TMU-35435, respectively, for 24 h (Figure 1A,B). Our results found that IR or TMU-35435 alone decreased the viability of cells in a dose- or concentration-dependent manner, respectively. Figure 1C shows the cell viability of cells treated with IR or TMU-35435 alone or in combination. The combined treatment significantly enhanced cytotoxicity compared with TMU-35435 or IR alone in 4T1 and MDA-MB-231 cells. The two TNBC cells were more sensitive to TMU-35435 and combined treatment in terms of cell viability as compared with the normal human mammary epithelial cells MCF-10A. Furthermore, the combination-index methods were used to confirm the observed synergism with TMU-35435 and IR combined therapy. The combination index (CI) of MDA-MB-231 and 4T1 cells was 0.5 and 0.808, respectively. Therefore, the combined treatment groups displayed synergistic cell killing effects. In addition, the survival curves of the clonogenic cell survival assays are shown in Figure 1D,E. The survival fractions of the TMU-35435 group (1 and 2 μM) markedly decreases compared to the IR group at 2, 4, 6, and 8 Gy. These results indicated that TMU-35435 treatment radiosensitized the TNBC cells.

### 2.2. Combination Treatment with TMU-35435 and IR Induces Misfolded Protein Aggregation, and TMU-35435 Inhibits the Interaction of HDAC6 with Dynein in 4T1 Cells

**Figure 2:**
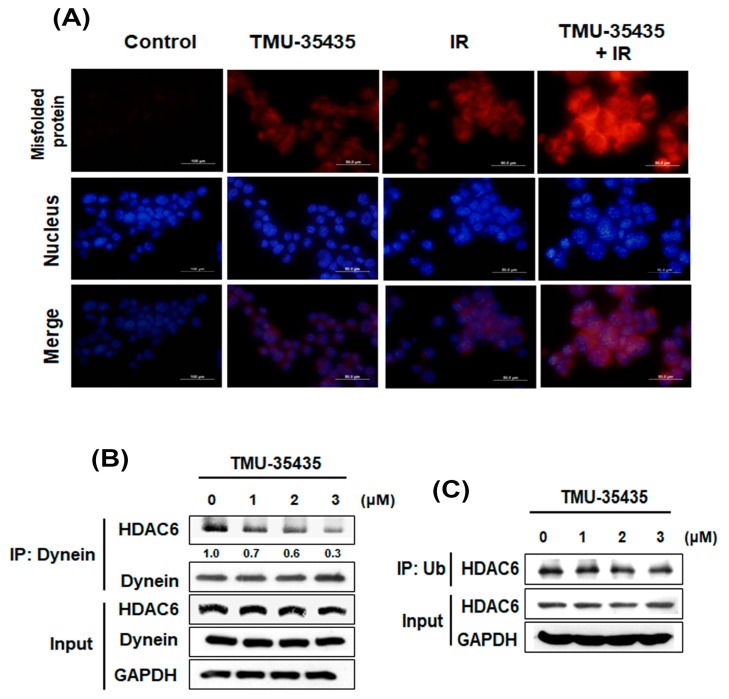
Misfolded protein aggregation and the interaction of HDAC6 with dynein and/or Ub in 4T1 cells treated with IR and TMU-35435. (**A**) The aggregation of the misfolded protein in 4T1 cells. The cells were treated with TMU-35435 (1 μM) and IR (4 Gy) for 24 h. The cells were stained with ProteoStat aggresome detection kit and Hoechst 33342. The red color and the blue color indicated aggregates and stained nuclei, respectively. Scale Bar: 50 μm. (**B**) 4T1 cells were cultured with TMU-35435 for 24 h. Whole-cell lysates were immunoprecipitated with an anti-dynein Ab. The immunoprecipitates were analyzed to Western blot analysis with an anti-HDAC6 Ab. (**C**) 4T1 cells were cultured with TMU-35435 for 24 h. Whole-cell lysates were immunoprecipitated with an anti-Ub Ab. The immunoprecipitates were analyzed to western blot analysis with an anti-HDAC6 Ab.

Recent studies have demonstrated that HDACis affect chaperone function and deregulate protein homeostasis. HDACi-mediated deregulation of chaperone function can induce protein misfolding and proteotoxic stress [25]. Another recent study concluded that IR increased misfolded protein by the generation of reactive oxygen species (ROS) [26]. Thus, we investigated whether combined treatment with TMU-35435 and IR could induce protein aggregation (Figure 2A). A ProteoStat aggresome detection kit was analyzed for the detection of protein aggregation. The red signals showed misfolded protein aggregates [27,28]. It was found that treatment with TMU-35435 or IR alone increased red signals in the cytoplasm. The combined treatment induced significant enhancement of protein aggregation compared with IR or TMU-35435 treatment alone. Previous studies have demonstrated that HDAC6 binds both dynein and polyubiquitinated proteins, thereby recruiting misfolded protein to dynein for transport to aggresomes along microtubules [29]. Therefore, we evaluated whether inhibition of HDAC6 activity by TMU-35435 changes the interaction of HDAC6 with ubiquitin (Ub) and/or dynein. After treatment with TMU-35435, IP of HDAC6 with dynein was significantly inhibited in a concentration-dependent manner in 4T1 cells (Figure 2B). However, immunoprecipitation of HDAC6 with Ub was unaffected (Figure 2C). Therefore, our results indicated that TMU-35435 suppressed the interaction of HDAC6 with dynein but did not alter the ubiquitinated HDAC6.

### 2.3. Measurement of Apoptosis and the Expression of ER Stress-Associated Proteins in Cells Treated with IR and TMU-35435 Separately or in Combination

**Figure 3:**
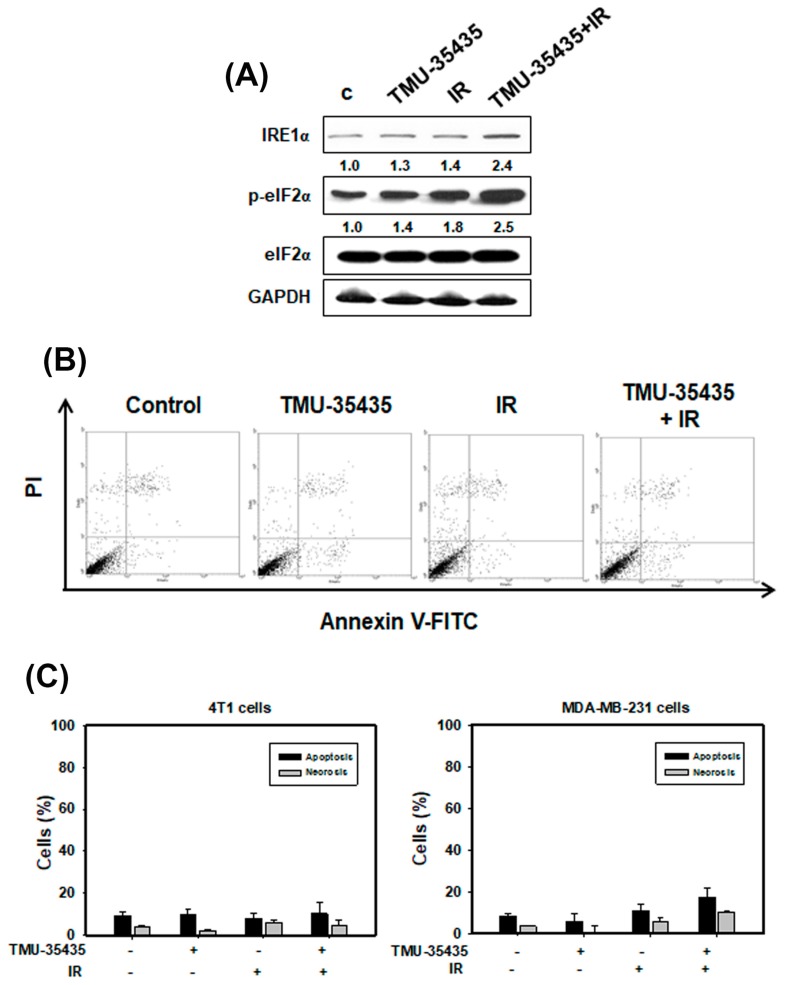
Measurement of endoplasmic reticulum (ER) stress and apoptosis in cells treated with different groups. (**A**) Effects of TMU-35435 and IR on the expression of ER stress-associated proteins. The cells were treated with IR (4 Gy) and TMU-35435 (1 μM) for 12 h. (**B**) Apoptosis was analyzed by an Annexin V apoptosis kit using flow cytometry. The cells were treated with TMU-35435 (1 μM) and IR (4 Gy) for 24 h. (**C**) Quantification of apoptosis and necrosis in MDA-MB-231 and 4T1 cells that received various treatments. The cells were treated with TMU-35435 (1 μM) and IR (4 Gy) for 24 h.

Recent evidence shows that IR-induced DNA damage causes ER stress and activates the UPR pathway [30]. Thus, to analyze the expression of ER stress-associated proteins, we used western blotting (Figure 3A). We found that phosphorylation of eIF2α and IRE1α increased with a combination treatment compared with IR or TMU-35435 alone. Therefore, the combined treatment caused ER stress. The accumulated evidence reveals that ER stress can cause apoptosis [8,11]. As shown in Figure 3B,C, apoptosis in MDA-MB-231 and 4T1 cells was analyzed using flow cytometry with an Annexin V/PI apoptosis kit. The quantitative results indicated that apoptotic and necrotic cells treated with TMU-35435 and/or IR for 24 h was low (Figure 3C). The observations are quite similar when compared to longer exposure time (48 h; Figure S1). Therefore, the combination treatment can induce ER stress but cannot trigger apoptosis.

### 2.4. Combined Treatment with IR and TMU-35435 Induces Autophagic Cell Death

**Figure 4:**
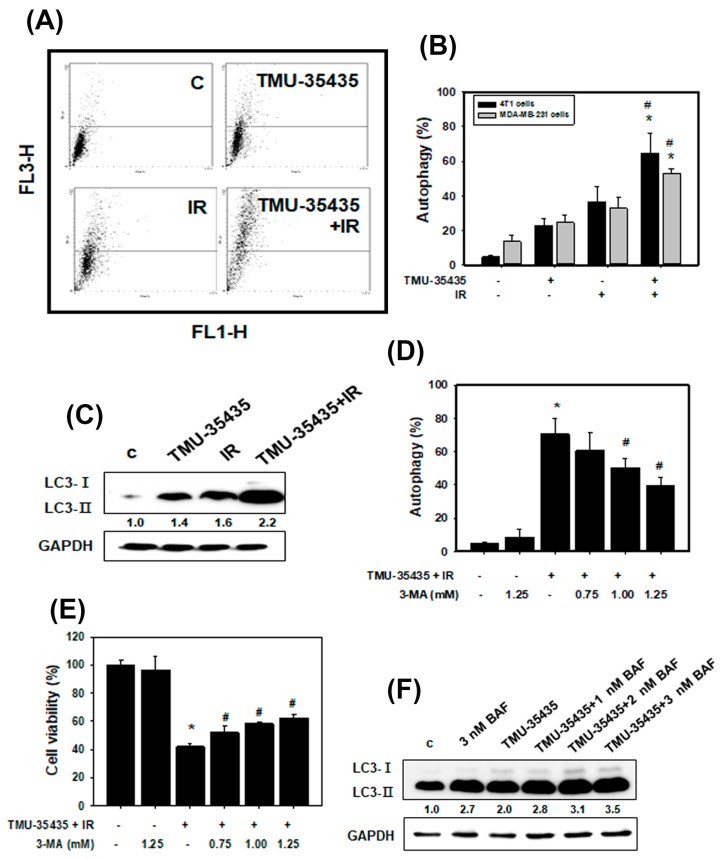
Combination treatment with TMU-35435 and IR causes autophagic cell death. (**A**) Measurement of acidic vesicular organelles (AVOs) in 4T1 cells by flow cytometry. The cells were treated with TMU-35435 (1 μM) and IR (4 Gy) for 24 h. (**B**) Quantification of AVOs in acridine orange (AO)-stained cells treated with TMU-35435 and IR separately or in combination by flow cytometry in MDA-MB-231 and 4T1 cells. * *p* < 0.05, TMU-35435 versus combination treatment. # *p* < 0.05, IR versus combination treatment. (**C**) LC3-I and II expression using Western blot analysis. (**D**) Quantification of AVOs in AO-stained cells pretreated with 3-MA by flow cytometry. 4T1 cells were pretreated with 3-MA for 1 h before receiving the combination treatment. ** *p* < 0.05, combination treatment versus control. # *p* < 0.05, combined treatment +3-MA versus combined treatment. (**E**) The cytotoxicity was measured after cells were incubated with or without 3-MA. * *p* < 0.05, combination treatment versus control. # *p* < 0.05, combined treatment +3-MA versus combined treatment. (**F**) Western blotting of LC3-I and LC3-II expression in 4T1 cells. The cells were pretreated with BAF for 1 h and then treated with TMU-35435 (1 μM) and IR (4 Gy) for 24 h.

We assessed type II programmed cell death (autophagy). Autophagy is featured by the formation of many acidic vesicles, which are called acidic vesicular organelles (AVOs) [31]. We quantified AVOs in acridine orange (AO)-stained cells by flow cytometry (Figure 4A,B). The combined treatment markedly increased AO-positive cells compared to those treated with IR or TMU-35435 alone in MDA-MB-231 and 4T1 cells. Microtubule-associated protein light chain 3 (LC3) has been widely used as an autophagic marker. The LC3-II expression is correlated with the number of autophagosomes [32]. Next, we detected LC3 conversion (LC3-I to LC3-II) using western blot analysis (Figure 4C). The expression level of the LC3-II protein was increased with the combined treatment, suggesting that the combination treatment produced autophagy in 4T1 cells.

**Figure 5:**
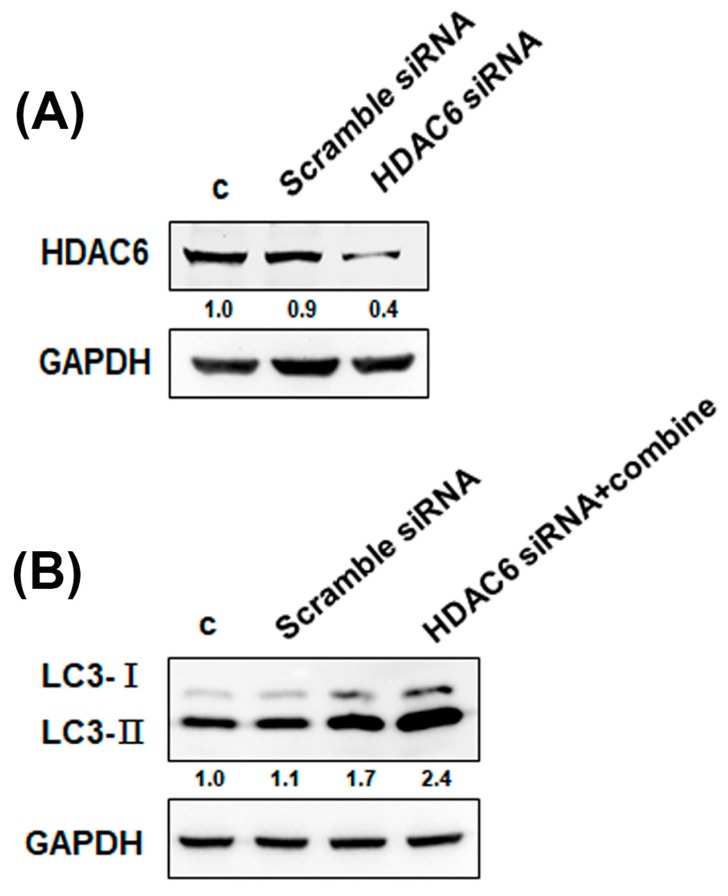
The inhibition of HDAC6 induces autophagy. (**A**) Transfection efficacy was confirmed by western blot analysis. The expression of HDAC6 protein in 4T1 cells transfected with control or HDAC6 small interfering RNA (siRNA) for 24 h. (**B**) Effects of HDAC6 siRNA on the expression of LC3 proteins. The cells were transfected with control or HDAC6 siRNA for 24 h. Then, 4T1 cells were treated with IR (4 Gy) and TMU-35435 (1 μM) for 24 h.

**Figure 6:**
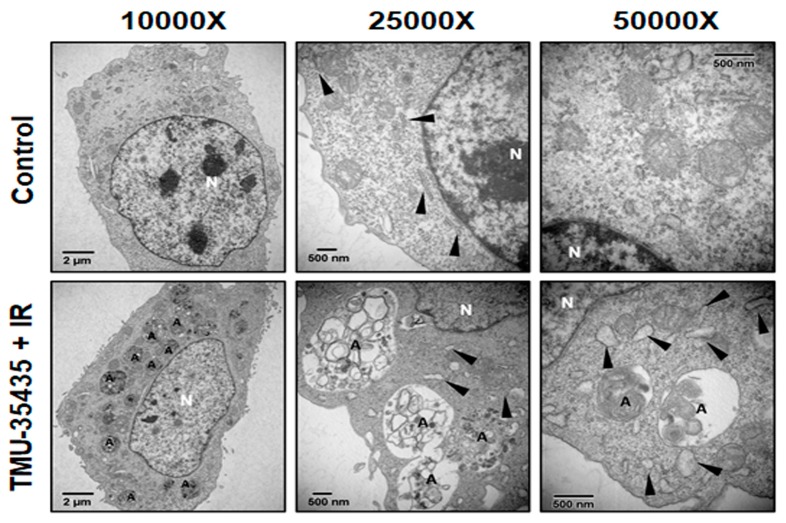
The ultrastructures of 4T1 cells treated with TMU-35435 (1 μM) and IR (4 Gy) for 24 h were observed using TEM. A, autophagic vacuoles. Black arrowheads, endoplasmic reticulum. N, nucleus.

To investigate whether regulation of the autophagic pathway has an influence on the survival of cells receiving the combined treatment, we analyzed the effects of 3-methyladenine (3-MA; an autophagy inhibitor) on cell viability. As shown in Figure 4D,E, 3-MA inhibited AVO formation and increased viability. Therefore, combination treatment-induced autophagy may lead to cell death. Evidence has been presented indicating that increases in autophagic markers may represent a raised generation of autophagosomes in autophagic flux and/or inhibition of autophagosomal maturation and degradation [33]. In addition, one of the standards for autophagic cell death is an increase in autophagic flux, not just an increase in autophagic markers [34]. Here, we examined whether the observed increases in autophagic markers in cells treated with bafilomycin A1 (BAF), which inhibits autophagosome-lysosome fusion, are due to produced autophagic flux. The results showed that the LC3-II expression was increased by the combination treatment with BAF (Figure 4F). Therefore, the combined treatment-induced accumulation of autophagic markers is not due to inhibition of autophagic degradation. We found that the combination treatment caused autophagic flux. Furthermore, we studied the relationship between HDAC6 and autophagy using HDAC6 small interfering RNA (siRNA). The expression of the HDAC6 proteins was obviously decreased in the cells treated with the HDAC6 siRNA compared with the scramble siRNA (Figure 5A). As shown in Figure 5B, transfection with HDAC6 siRNA significantly enhanced the expression of LC3-II of combined treatment in 4T1 cells. In addition, we analyzed the ultrastructure of the 4T1 cells using electron microscopy. TEM of 4T1 cells that received the combined treatment revealed an increase in the number of autophagic vacuoles (Figure 6). Additionally, there were more numerous dilations of the ER membranes, which is a sign of ER stress, in the combined treatment group than in the control group (Figure 6).

### 2.5. Combination Treatment Inhibits Tumor Growth through the Induction of ER Stress and Autophagy in a Mouse Model of Orthotopic Breast Cancer 

**Figure 7:**
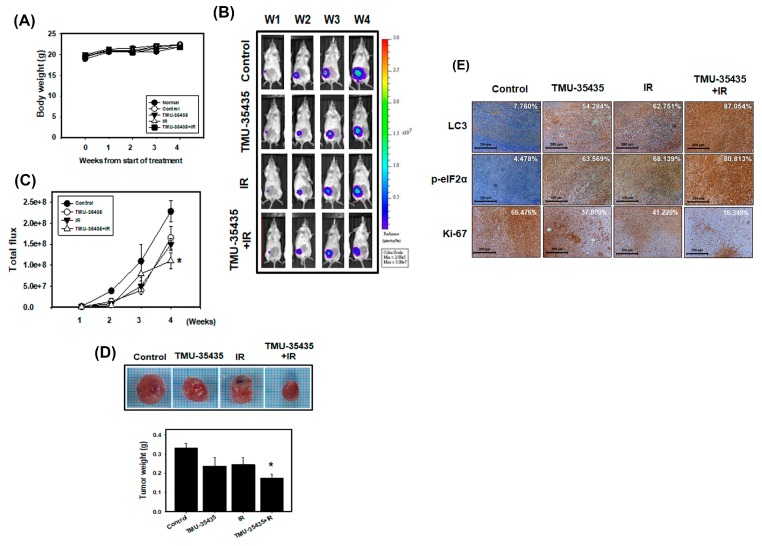
Combined treatment with TMU-35435 and IR increases antitumor effects in a mouse model of orthotopic breast cancer. (**A**) Measurement of body weight in Balb/c mice was evaluated once per week. (**B**) 4T1-Luc cells were injected into Balb/c mice of the mammary fat pads, which were analyzed for luciferase signals by an in vivo imaging system (IVIS) 200. (**C**) Quantification of the luciferase signals. (**D**) The tumor weight was measured in the Balb/c mice after sacrifice. * *p* < 0.05 versus control. (**E**) Immunohistochemistry (IHC) staining of orthotopic tumor tissues. The LC3 and p-eIF2α expression was determined by IHC staining. The percentage of positive cells was analyzed by HistoQuest software (TissueGnostics). Scale Bar: 200 μm.

To establish the orthotopic breast cancer model, 4T1 cells were stably transfected with luciferase. 4T1-Luc cells were then implanted in the mammary fat pads of Balb/c mice. The animals were observed for 4 weeks following treatment with IR and TMU-35435 separately or in combination, after which their body weights were determined and biochemical examinations were performed. As shown in Figure 7A, none of the treatment produced any overt abnormalities or losses of body weight. Moreover, no detectable toxicity was obvious upon biochemical tests following treatment with TMU-35435 and/or IR alone (Table 1). Furthermore, we assessed the therapeutic effect of IR and TMU-35435 alone or in combination on the growth of orthotopically implanted 4T1 cells in mice. The in vivo imaging system (IVIS) imaging was analyzed after cell implantation every week (Figure 7B,C). The bioluminescence imaging showed a gradual increase in tumor volume in the control group. The combined treatment group had lower tumor volumes than the control group (*p* < 0.01). The tumor weight in the combination treatment was significantly decreased compared to the control (*p* < 0.01; Figure 7D). Next, the expression patterns of LC3 and the phosphorylation of eIF2α in the 4T1 tumors were analyzed by immunohistochemistry (IHC) staining (Figure 7E). LC3 expression in tumor tissue in the TMU-35435, IR, and combination groups was markedly higher than that in the control group. Similarly, when the phosphorylation of eIF2α was examined, we found that TMU-35435 treatment, IR treatment, and the combination treatment enhanced the phosphorylation of eIF2α expression compared to the control treatment. The proliferation marker Ki-67 in the tumor tissue of combination groups decreased obviously. These results of animal model indicated that the combination of TMU-35435 and IR inhibited tumor growth through the induction of ER stress and autophagy in a mouse model of orthotopic breast cancer.

## 3. Discussion

HDAC6 expression has been found to be high in several cancers, including breast cancer [25,35]. Previous research indicated that cancer cells treated with a pan-HDACi induce hyperacetylation of hsp90 to inhibit its chaperone function [25,36]. Cellular chaperones promote the folding and maturation of newly synthesized proteins and prevent the aggregation of misfolded proteins [25]. Studies to identify the HDAC isoform revealed that the predominantly cytosolic isoform HDAC6 was the major hsp90 deacetylase [37]. If HDAC6 is lacking, cells cannot clear cytoplasmic misfolded/unfolded protein aggregates and cannot appropriately form aggresomes. The resulting misfolded/unfolded protein accumulation leads to cellular stress [15]. In addition, IR has been reported to increase protein misfolding through the generation of ROS [26]. In this study, TMU-35435 or IR alone caused misfolded protein aggregation in the cytoplasm. Marked enhancement of protein aggregation was found in the combination treatment group compared to TMU-35435 treatment group or the IR treatment group (Figure 2A). Previously, the authors reported that HDAC6 binds both dynein and polyubiquitinated proteins for transport to aggresomes along microtubules [29]. Therefore, HDAC6 plays a main role in the clearance of misfolded or unfolded proteins in the cytoplasm. We found that inhibition of HDAC6 activity by TMU-35435 altered the interaction between dynein and HDAC6 (Figure 2B), suppressing the clearance of misfolded proteins and inhibiting aggresome formation. Evidence has been presented indicating that the accumulation of misfolded proteins in the ER induces ER stress and activates the UPR pathway [38]. Masud et al. indicated that an unresolved UPR and protracted ER stress result in sustained activation of C/EBP homologous protein (CHOP) induction and eIF2α phosphorylation, resulting in ER stress-induced apoptosis that involves where the activity of caspase pathways is involved [39]. HDACi induced G2/M arrest and intrinsic/mitochondrial-mediated apoptosis in non-small-cell lung cancer cells [23]. Our data found that IRE1α expression and the phosphorylation of eIF2α increased after combination treatment (Figure 3A). In the orthotopic model of breast cancer, combination treatment significantly increased eIF2α phosphorylation in tumor tissue (Figure 7E). However, the occurrence of apoptosis and necrosis in MDA-MB-231 and 4T1 cells receiving the combined treatment was low (Figure 3B,C). One of the reasons for this could be that TNBC cells overexpress antiapoptotic proteins, leading to resistance to apoptosis [40].

Previous studies have shown that stress caused by the accumulation of protein aggregates in the cytoplasm can mediate autophagy [41]. Furthermore, ER stress induces autophagy [38]. It has been reported that SAHA, a histone deacetylase inhibitor, stimulates autophagy by the inhibition of the Akt/mTOR pathway in TNBC [42]. SAHA induced autophagy and restrained cell viability through reducing survivin and XIAP protein stability in human breast cancer cells [43]. In addition, IR induces autophagy by regulating the DNA damage repair-related protein PARP-1 [44]. In our current study, the observations were quite similar to those of other investigators. We found a significant increase in autophagic cells in the combined treatment group (Figure 4A,B). The expression level of LC3-II protein increased with combined treatment (Figure 4C). TMU-35435 treatment, IR treatment, and combination treatment significantly induced autophagy in tumor tissue (Figure 7E). The results suggest that the combination treatment caused autophagy in TNBC. However, the role of autophagy in controlling cancer survival or cell death is debatable. Zhou et al. found that the inhibition of autophagy can significantly increase radiosensitivity in nasopharyngeal carcinoma cells [44]. Our previous study indicated that autophagy plays a cytotoxic role in TNBC cells receiving combined IR and HDACi treatment [45]. HDACis can induce cell cycle arrest and often concomitantly with senescence [46]. Autophagy is identified as a new effecter mechanism of senescence [47]. However, some HDACis could promote the expansion of the tumor initiating subpopulation through reprogramming of differentiated cancer cells into stem-like cells [48]. In this study, we further revealed that the combination treatment triggered autophagic flux and autophagic cell death (Figure 4E,F).

HDACs participate in the DNA repair pathway and regulate the expression of genes related to this process [49]. Data accumulated by us and others have demonstrated that HDACis restrained DNA repair through the downregulating or inhibiting the activity of DNA repair proteins, including homologous recombination (HR) and non-homologous end joining (NHEJ) pathways, in cancer cells [50,51]. Therefore, HDACis could serve as radiosensitizers. Our previous study indicated that combined treatment with IR and HDACi induced stronger cytotoxicity in TNBC than either treatment alone. The HDACi may contribute to this combined effect because the HDACi inhibited IR-induced DNA repair pathways [51]. Our current study showed that treatment with TMU-35435 strongly increased the cytotoxic effect exerted by IR on the viability of TNBC cells (Figure 1). In our orthotopic breast cancer of an animal model, TMU-35435 plus IR had better efficacy than the individual treatments in delaying the growth of tumors (Figure 7). Numerous reports in the literature have shown that autophagy signaling is a critical target to improve anticancer therapy. Chaachouay et al. reported that radioresistant breast cancer cells show a strong post-IR induction of autophagy, which then serves as a protective role [52]. Another study demonstrated that treatment with an Akt inhibitor reduced cell viability and radiosensitizing the cells through autophagy induction in glioma cells [53]. Nevertheless, whether inhibition or overactivation of autophagy is more beneficial, is still being disputed.

## 4. Materials and Methods

### 4.1. Culture and Cell Viability Assay

The murine breast cancer cell line 4T1 (CRL-2539), human breast cancer cell line MDA-MB-231 (HTB-26), and human mammary epithelial cell line MCF-10A (CRL-10317) were obtained from the American Type Culture Collection (ATCC). The luciferase-expressing 4T1-Luc was obtained from Prof. Yi-Ching Wang (Department of Pharmacology, National Cheng Kung University, Tainan, Taiwan). The 4T1 and MDA-MB-231cells were maintained in Dulbecco’s Modified Eagle’s Medium (DMEM; Gibco BRL, Grand Island, NY, USA) with 10% fetal bovine serum (Caisson Labs, Logan, UT, USA) and antibiotics containing 100 U/mL penicillin and 100 mg/mL streptomycin (Gibco BRL). The MCF-10A were maintained in DMEM/F12 supplemented with 10 µg/mL insulin, non-essential amino acid, 0.5 µg/mL hydrocortisol, 20 ng/mL epidermal growth factor, 10% fetal bovine serum, and antibiotics (Gibco BRL). All cells were incubated in a humidified atmosphere containing 5% CO_2_ at 37 °C. For the cell viability assay, treated cells were harvested and resuspended in phosphate-buffered saline (PBS), and then 20 μL of each cell suspension was mixed with an equal amount of trypan blue solution. The mixture was placed on a hemocytometer, and there were no blue-stained cells counted as alive cells.

### 4.2. Drug and IR Treatment

The novel HDACi TMU-35435, *N*-hydroxy-6-(5-methyl-4-acridinecarbamoyl) hexanamide, was obtained from Dr. W. J. Huang (Graduate Institute of Pharmacognosy, Taipei Medical University, Taipei, Taiwan) and requests for this compound should be sent to wjhuang@tmu.edu.tw. IR was performed with 6 MV X-rays using a linear accelerator (Digital M Mevatron Accelerator, Siemens Medical Systems, CA, USA) at a dose rate of 5 Gy/min. An additional 10 cm of tissue-equivalent material was placed under the flasks to obtain full backscatter, and 2 cm of a tissue-equivalent bolus was placed on the top of the plastic tissue-culture flasks to ensure electronic equilibrium. In the combined group, cells were treated with TMU-35435 immediately after IR treatment.

### 4.3. Drug Interaction Analysis

The effect of the combination treatment was assessed by the combination index (CI) method using CalcuSyn software (Biosoft,Cambridge, UK). The data of cell viability were entered into the CalcuSyn software and CI values were calculated. CI < 1, CI = 1, and CI > 1 indicate synergism, additive effect, and antagonism, respectively.

### 4.4. Clonogenic Assay

Cells were irradiated with 2, 4, 6, or 8 Gy. TMU-35435 was added to 4T1 cells at concentrations of 1 or 2 μM. The cells were trypsinized and counted. Cells were then cultured on six-well plates to allow for colony development. After 10 days, colonies (defined as a colony with ≥50 cells) were stained with crystal violet solution. Plating efficiency (PE) is the ratio of the number of colonies to the number of cells seeded in the non-irradiated group. Calculation of surviving fractions (SFs) was performed using the equation SF = colonies counted/(cells seeded × PE), taking into consideration the individual PE.

### 4.5. Misfolded Protein Detection

Misfolded protein in cells was observed with ProteoStat Aggresome Detection Kit (Enzo Life Sciences, Farmingdale, N.Y. USA). Cells were seeded directly on glass slides. After treatment, the slides were incubated with 4% formaldehyde at room temperature for 30 min, and covered with permeabilizing solution (0.5% Triton X−100, 3 mM EDTA, pH 8) on ice for 30 min. Next, the cells were stained with a dual detection reagent containing Hoechst 33342 (nuclear stain) and ProteoStat aggresome detection reagent and incubated for 30 min at room temperature. After removing excess buffer and placing coverslips on the microscope slides, the stained cells were analyzed for aggresome signals and nuclear signals by fluorescence microscopy (Olympus, Japan).

### 4.6. Autophagy and Apoptosis Detections

Autophagy was analyzed by staining with the cells 1 μg/mL acridine orange (AO) (Sigma-Aldrich, MO, USA) for 20 min. Then acidic vesicular organelles (AVOs), a characteristic of autophagy, were quantified using flow cytometry (BD Biosciences, San Jose, CA, USA). AO-stained cells were analyzed using FL3 mode (>650 nm) to value the bright red fluorescence and FL1 mode (500–550 nm) to value the green fluorescence. In addition, an Annexin V/PI detection kit (Calbiochem, CA, USA) was used to observe the translocation of phosphatidyl serine to the cell surface. Harvested cells were incubated with 1× Annexin V-binding buffer containing Annexin V-FITC and/or propidium iodide (PI), and the labeled cells were collected by flow cytometry (BD Biosciences, San Jose, CA, USA).

### 4.7. Western Blotting and Immunoprecipitation (IP)-Western Blotting

Cells were lysed in golden lysis buffer at 4 °C for 1 h. Proteins isolated from the cells and HR Pre-Stained Protein Marker 10–170 kDa (BIOTOOLS, Taiwan) were loaded, separated by SDS-PAGE, and transferred to polyvinylidene fluoride (PVDF) membranes (Thermo Fisher Scientific, Rockford, IL, USA), and the membranes were then blocked with skim milk. Anti-GAPDH, anti-IRE1α, and anti-phospho-eIF2α antibodies were obtained from Abcam (Cambridge, MA, USA); an anti-HDAC6 antibody was obtained from Cayman (Ann Arbor, MI, USA); anti-dynein antibody was obtained from Merck Millipore (Darmstadt, Germany); anti-Beclin 1 antibody was obtained from Cell Signaling Technology (Ipswich, MA, USA); anti-LC3 and anti-eIF2α antibodies were obtained from Abgent (San Diego, CA, USA); and an anti-p62/SQSTM1 antibody was obtained from MBL (Nagoya, Japan). The membranes were incubated with primary antibody solution at 4 °C overnight. After washing, the membranes were incubated with horseradish peroxidase (HRP)-conjugated secondary antibody for 1 h. Then, signals on membranes were detected by immobilon Western Chemiluminescent HRP Substrate (Merck Millipore, Darmstadt, Germany) and exposed to X-ray film (Fuji medical X-ray film, Japan). For IP western blotting, whole-cell protein lysates were premixed with primary antibodies in immunoprecipitation buffer overnight at 4 °C, incubated with protein G plus/protein agarose (Merck Millipore, Darmstadt, Germany) for 1 h. After washing three times, the beads were resuspended in 4× sample dye and boiled at 95 °C for 10 min. The supernatant was subjected to western blotting as described above. Raw data of Western blot is shown in Figure S2.

### 4.8. RNA Interference (RNAi)

Cells were seeded overnight with or without HDAC6 siRNA (4390771, Thermo Fisher Scientific, Waltham, MA, USA) according to Mirus transfection protocol (TransIT-X2^®^, Mirus, Madison, WI, USA). Briefly, OptiMEM (Invitrogen) mixed TransIT-X2 reagents and siRNA. Then, the complex mixture was incubated for 30 min and placed drop-to-drop in each well for 24 h. 

### 4.9. Transmission Electron Microscopy

Treated cells were harvested and fixed in 0.1 M cacodylate buffer containing 2.5% glutaraldehyde and 2% paraformaldehyde at 4 °C overnight and then postfixed in fixation buffer containing 1% OsO_4_ for 1 h. Then, ultrathin sections were observed using a transmission electron microscope (JEOL JEM-1200EX, Japan).

### 4.10. In Vivo Orthotopic Breast Cancer Model

All mice experiments were handled in accordance with the guidelines in the Guide for Care and Use of Laboratory Animals, Medical College, National Cheng Kung University (IACUC Approval Number: 105161). Six-week-old female Balb/c mice were acquired from the National Laboratory Animal Center (Taiwan) and housed at 23 ± 2 °C with 60% ± 5% relative humidity and subjected to a 12-h light/12-h dark cycle. 4T1-Luc cells (5 × 10^4^ cells) were injected into the 4th mammary fat pads of lactiferous ducts the in mice. One week post injection, the mice were randomized into five groups (*n* = 5 for each group): (1) the control, which was intraperitoneally (ip) injected with DMSO, (2) the TMU-35435 group: which was i.p. injected with 15 mg/kg TMU-35435 three times per week for three weeks, (3) the IR group, which was given a single dose of 4 Gy IR, and (4) the TMU-35435 + IR group, which was given a combination therapy of 15 mg/kg TMU-35435 three times per week and a single dose of 4 Gy IR at the beginning of the first week. Mouse body weight, one of the parameters for evaluating the systemic toxicity of the treatments, was measured once per week. In vivo bioluminescence imaging of tumors was obtained through an IVIS 200 system containing a data acquisition computer running Living Image Software (Xenogen, Alameda, CA, USA). Before imaging, the mice were anesthetized with 1%–4% isoflurane and injected with 150 mg/kg VivoGlo Luciferin (Promega, Madison, WI, USA). Mice were sacrificed via CO_2_, and tumor tissues and serum were collected for immunohistochemistry and biochemical testing with a FUJI DRI-CHEM 4000i machine and FUJI DRI-CHEM Slides (Fujifilm, Tokyo, Japan).

### 4.11. Biochemistry Test

Whole blood samples of experimental mice were collected by intracardiac puncture into tube and centrifuged at 2000× *g* for 20 min. Biochemistry evaluation included glutamate pyruvate transaminase (GPT), glutamate oxaloacetate transaminase (GOT), creatinine, blood urea nitrogen (BUN), and albumin. Statistical analysis was performed using a Student’s *t*-test.

### 4.12. Immunohistochemical (IHC) Staining Analysis

After formalin fixation and paraffin embedding, tumor sections were deparaffinized in xylene and rehydrated through graded concentrations of ethanol in water. Following microwave treatment in sodium citrate buffer to retrieve antigens, the slides were soaked in 3% H_2_O_2_/methanol for 10 min to block the activity of endogenous peroxidase. After the washing process, the samples were incubated with anti-LC3, anti-phospho-eIF2α and Ki-67 (Cell Signaling Technology, Ipswich, MA, USA) antibodies, and a Starr Trek Universal HRP detection kit (Biocare Medical, Concord, CA, USA) was then used to detect the antibodies. Finally, the slides were stained with hematoxylin and coverslipped with Entellan New (Merck Millipore, Darmstadt, Germany). IHC images were taken at low magnification (100×) and analyzed using HistoQuest software (V.4.0.4.0158, TissueGnostics, Vienna, Austria).

### 4.13. Statistical Analysis

The data are presented as the mean ± standard deviation (SD) of the results of at least three independent experiments. Statistical significance was analyzed with two-tailed paired Student’s *t*-tests or one-way analysis of variance with post hoc Dunnett’s test. A *p*-value < 0.05 (*p* < 0.05) was statistically significant.

## 5. Conclusions

**Figure 8:**
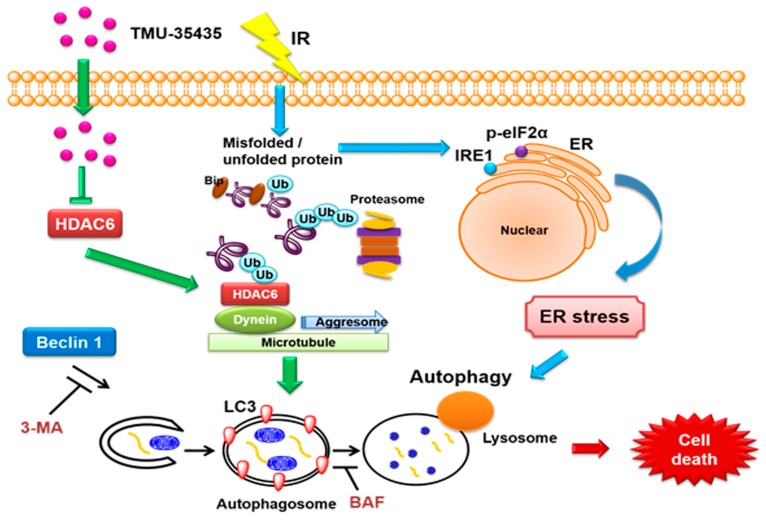
TMU-35435 enhances radiation sensitivity through the induction of misfolded protein aggregation and autophagic cell death in triple-negative breast cancer (TNBC). IR causes the aggregation of misfolded protein and ER stress. TMU-35435 suppresses the interaction of HDAC6 with dynein and then causes misfolded protein aggregation. Furthermore, combined treatment-induced ER stress can cause autophagic cell death.

Given our data and the existing evidence, we can construct an integrated network of protein aggregation, ER stress and autophagy, as shown in the schematic diagram in Figure 8. Our results overall suggested that significantly enhanced cytotoxicity was exerted by the combined treatment compared with IR or TMU-35435 alone in TNBC cells. Moreover, TMU-35435 suppressed the interaction of HDAC6 with dynein. Therefore, significant enhancement of protein aggregation and ER stress was found in the combination treatment. Combined IR and TMU-35435 treatment increased autophagic flux and autophagic cell death. In the mouse model of orthotopic breast cancer, tumor growth was suppressed by combination treatment with IR and TMU-35435 through the induction of ER stress and autophagy. Our current study indicated that TMU-35435 could serve as a radiosensitizer against TNBC. 

## Figures and Tables

**Table 1:** Biochemistry tests including GOT, GPT, albumin, blood urea nitrogen (BUN), and creatinine.

Item/Group	Normal	4T1 Cells			
		Control	TMU-35435	IR	TMU-35435 + IR
**GOT (U/L)**	170.20 ± 23.87	186.40 ± 26.90	153.20 ± 12.49	140.80 ± 10.84	158.20 ± 15.94
**GPT (U/L)**	43.40 ± 4.06	33.00 ± 1.41	38.60 ± 3.78	34.80 ± 1.11	36.00 ± 1.38
**Albumin (g/dL)**	1.80 ± 0.03	1.72 ±0.08	1.62 ± 0.07	1.78 ± 0.06	1.76 ± 0.07
**BUN (mg/dL)**	24.52 ± 0.40	24.52 ± 1.26	22.92 ± 0.77	23.36 ± 1.41	22.82 ± 1.57
**Creatinine (mg/dL)**	0.24 ± 0.02	0.26 ± 0.02	0.28 ± 0.02	0.26 ± 0.02	0.22 ± 0.02

## Supplementary Materials

The following are available online at https://www.mdpi.com/2072-6694/11/11/1703/s1, Figure S1: Measurement of apoptosis in 4T1 cells treated with different groups, Figure S2: Raw data of Western blot.
