# Supplementary material for: A New Histone Deacetylase Inhibitor Enhances Radiation Sensitivity through the Induction of Misfolded Protein Aggregation and Autophagy in Triple-Negative Breast Cancer
Source: Cancers (Basel). 2019 Nov 1;11(11):1703. doi: 10.3390/cancers11111703 (PMC6896096; doi:10.3390/cancers11111703)
Supplement: Supplementary file 1 [file cancers-11-01703-s001.pdf]

Supplementary Materials: A New Histone Deacetylase Inhibitor Enhances Radiation Sensitivity through the Induction of Misfolded Protein Aggregation and Autophagy in Triple-negative Breast Cancer

Hui-Wen Chiu, Ya-Ling Yeh, Sheng-Yow Ho, Yuan-Hua Wu, Bour-Jr Wang, Wei-Jan Huang, Yuan-Soon Ho, Ying-Jan Wang, Li-Ching Chen and Shih-Hsin Tu

(A)

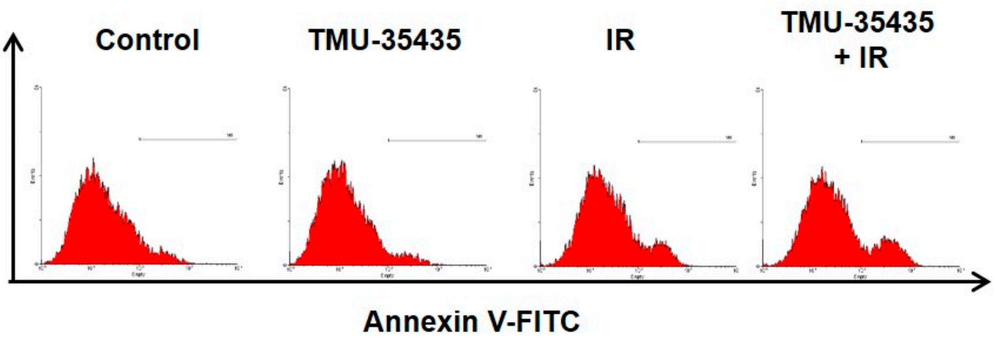

(B)

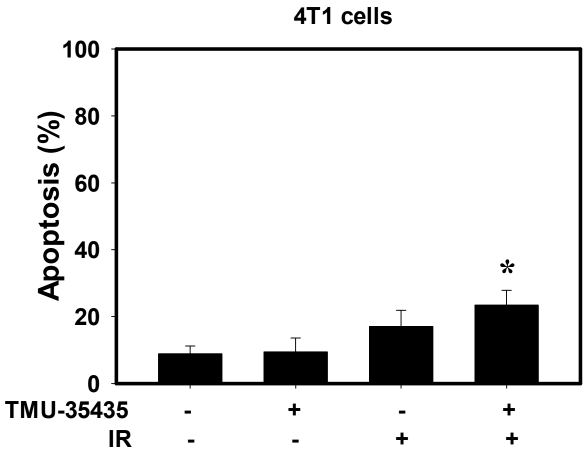

Figure S1. Measurement of apoptosis in 4T1 cells treated with different groups. (**A**) Apoptosis was analyzed by an Annexin V apoptosis kit using flow cytometry. The cells were treated with TMU-35435 (1 μM) and IR (4 Gy) for 48 h. (**B**) Quantification of apoptosis and necrosis in 4T1 cells that received various treatments. The cells were treated with TMU-35435 (1 μM) and IR (4 Gy) for 48 h. *$p < 0.05$, TMU-35435 versus combination treatment.

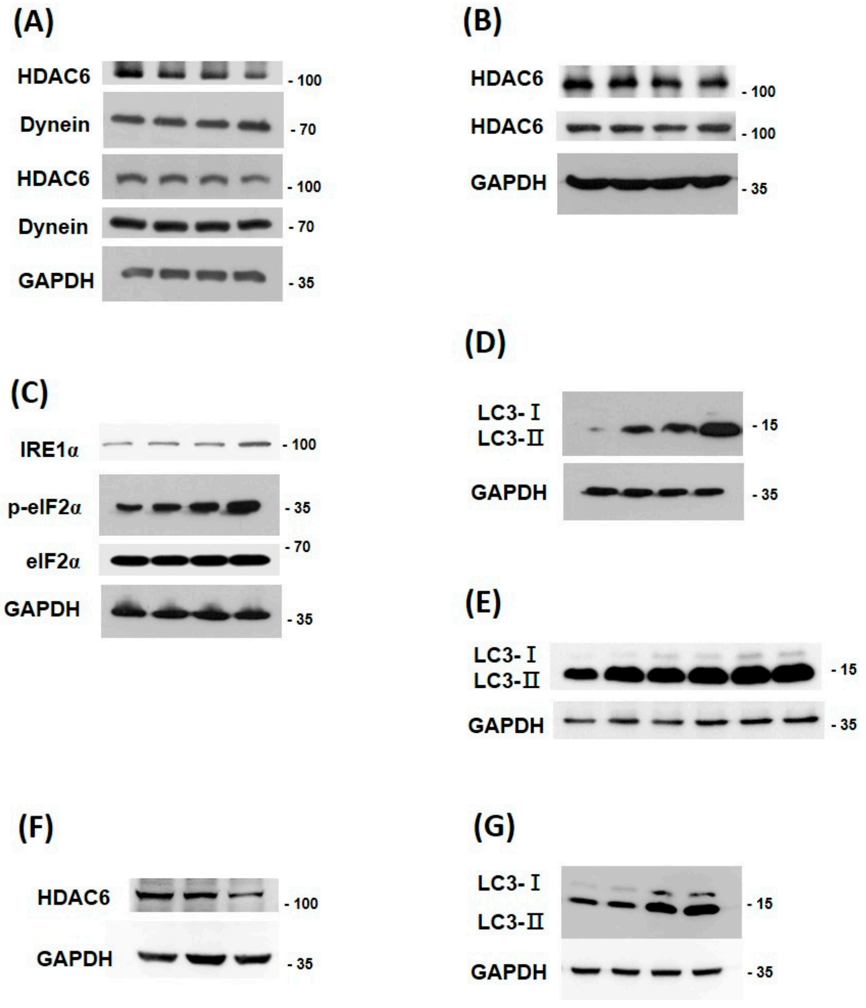

Figure S2. Raw data of Western blot. (**A**) Raw data for Figure 2B. (**B**) Raw data for Figure 2C. (**C**) Raw data for Figure 3A. (**D**) Raw data for Figure 4C. (**E**) Raw data for Figure 4F. (**F**) Raw data for Figure 5A. (**G**) Raw data for Figure 5B.

© 2019 by the authors. Licensee MDPI, Basel, Switzerland. This article is an open access article distributed under the terms and conditions of the Creative Commons Attribution (CC BY) license (http://creativecommons.org/licenses/by/4.0/).
